# Supplementary figures and images for: IGF2BP1 induces neuroblastoma via a druggable feedforward loop with MYCN promoting 17q oncogene expression
Source: Mol Cancer. 2023 May 29;22:88. doi: 10.1186/s12943-023-01792-0 (PMC10226260; doi:10.1186/s12943-023-01792-0)

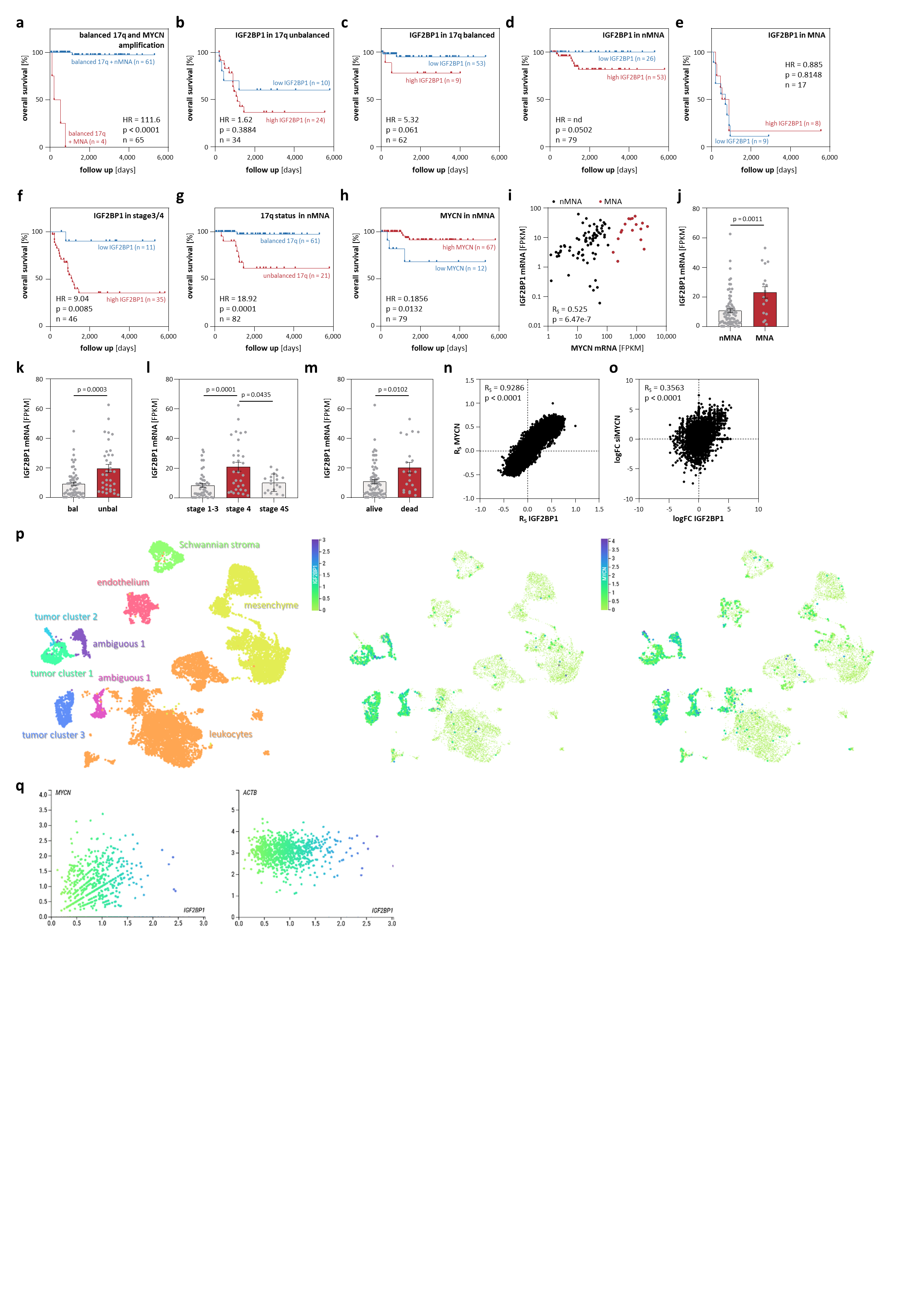

Supplement: Supplementary file 1 — Additional file 1: Supplementary Fig. 1. IGF2BP1 and MYCN upregulation synergize in high-risk neuroblastoma. (a-h) Kaplan-Meier survival analyses by indicated conditions (best cut-off). (i) Spearman correlation of IGF2BP1 and MYCN mRNA expression in neuroblastoma tumors. MNA samples are color-coded in red. (j-m) IGF2BP1 mRNA expression separated by MYCN status (j), Chr 17 balance status (k), INSS stage (l) and survival (m). (n) Correlation analysis of Spearman correlation coefficients determined for IGF2BP1- and MYCN-associated gene expression in primary tumors. (o) Spearman correlation analysis of the logFC of mRNAs after IGF2BP1- or MYCN-knockdown in BE(2)-C. (p) Expression of IGF2BP1 (middle) and MYCN (right) in neuroblastoma upon single cell sequencing. Cell types are indicated in the left panel. (q) Correlation analysis of single cell sequencing data of IGF2BP1 and MYCN (left) or ACTB (right). nMNA - MYCN non-amplified, MNA - MYCN-amplified, bal - balanced Chr 17, unbal - unbalanced Chr 17. [file 12943_2023_1792_MOESM1_ESM.tif]

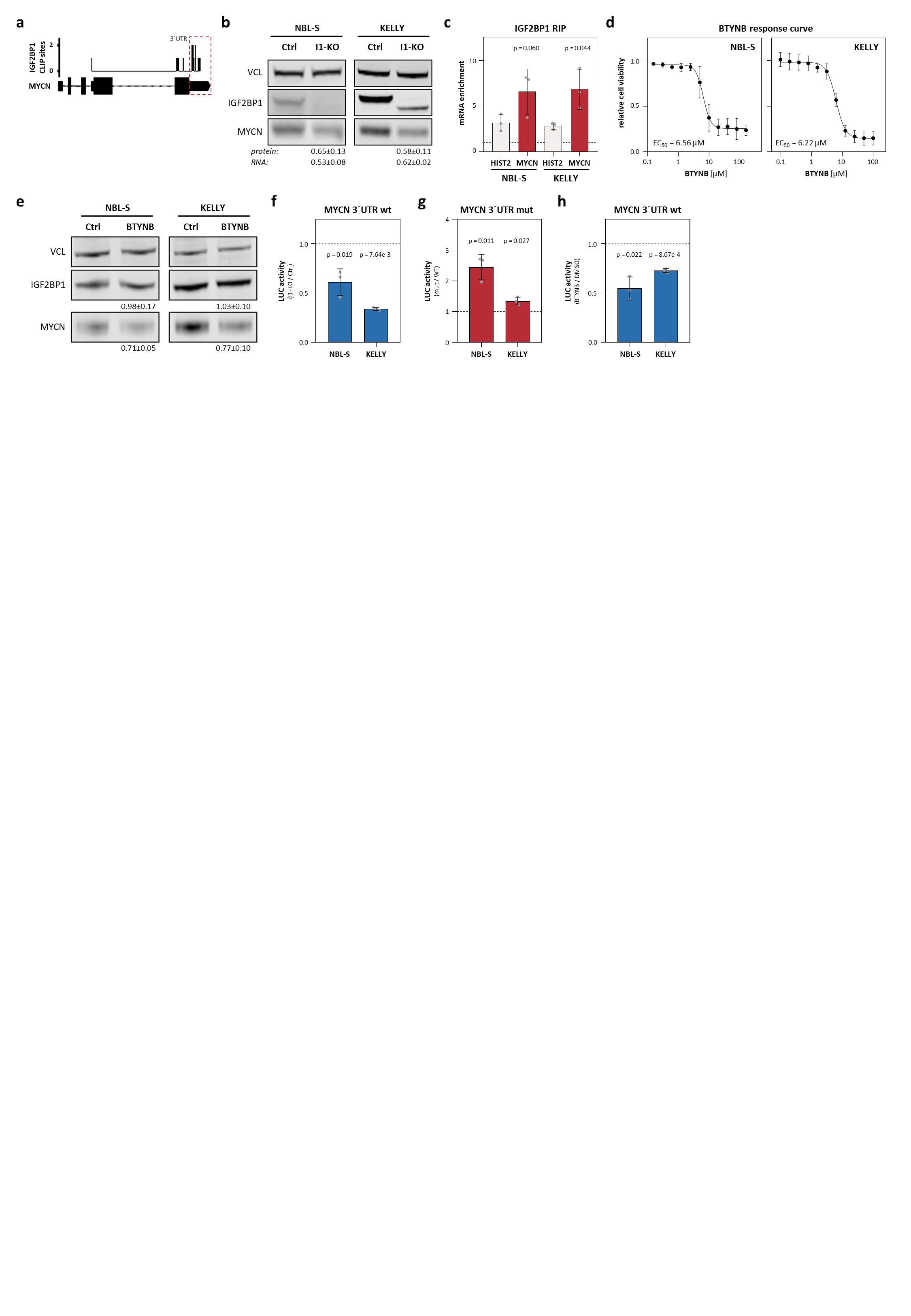

Supplement: Supplementary file 2 — Additional file 2: Supplementary Fig. 2. IGF2BP1-driven and BTYNB-druggable MYCN expression is conserved in NBL-S and KELLY neuroblastoma cells. (a) IGF2BP1-CLIP hits in the MYCN mRNA derived from two studies in hESC. (b) Western blot and RT-qPCR analyses of MYCN expression upon I1-KO in NBL-S and KELLY (n = 3). (c) IGF2BP1-RIP analyses in NBL-S and KELLY (n = 3). (d) BTYNB-response curves in NBL-S and KELLY (n = 3). (e) Western blot analysis of MYCN expression upon treatment of NBL-S and KELLY with BTYNB (n = 3). (f-h) Relative activity of MYCN 3´UTR luciferase reporter in NBL-S and KELLY determined between I1-KO and control cells (f), mutant and wildtype MYCN 3´UTR (g) or BTYNB- and DMSO-treated cells (h; all n = 3). [file 12943_2023_1792_MOESM2_ESM.tif]

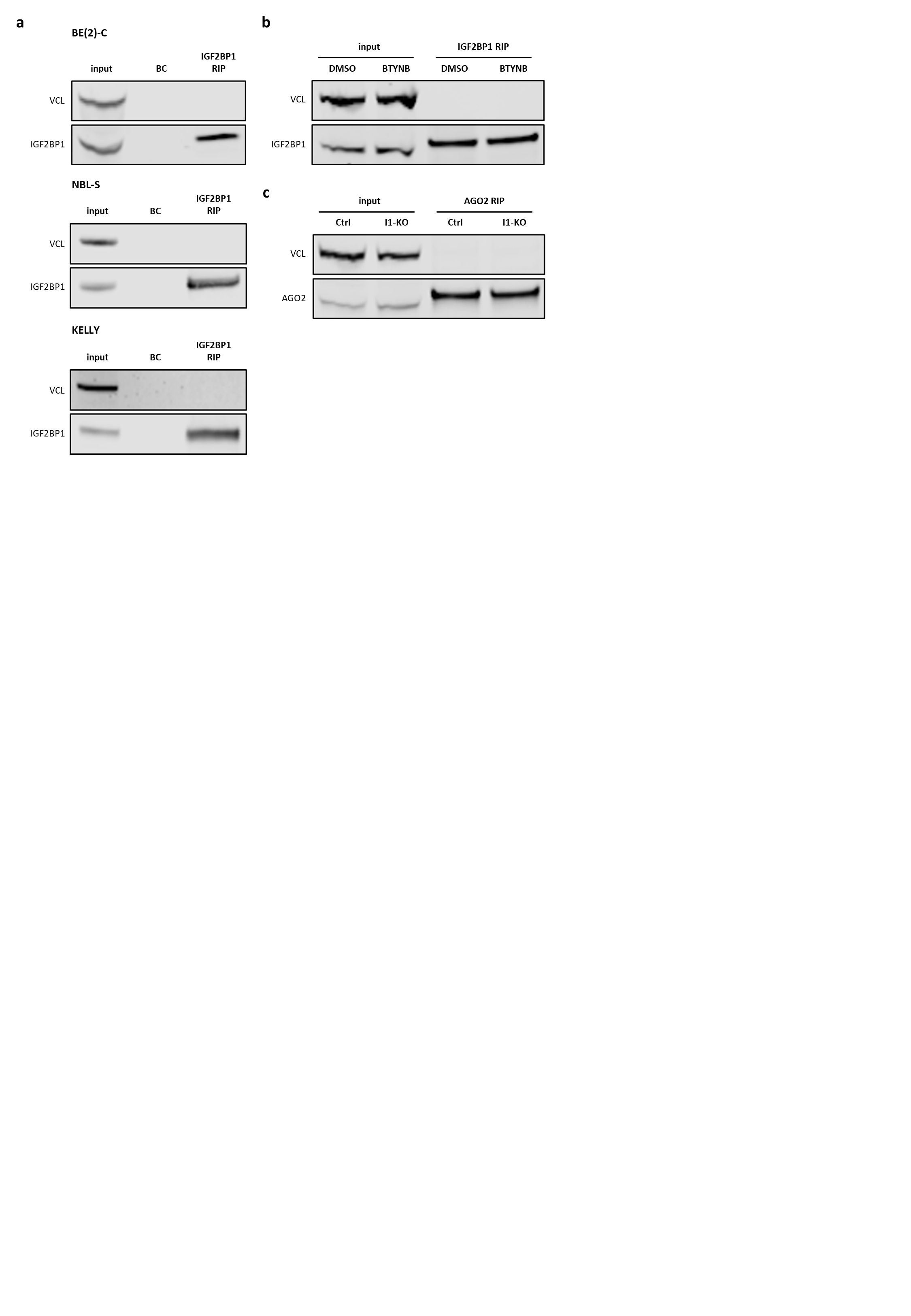

Supplement: Supplementary file 3 — Additional file 3: Supplementary Fig. 3. Validation of IGF2BP1 and AGO2 immunoprecipitation. (a-c) Isolation of indicated proteins (IGF2BP1, AGO2) in RIP studies from parental BE(2)-C, NBL-S and KELLY (a), DMSO- and BTYNB-treated BE(2)-C (b) or parental (Ctrl) and I1-KO (c) cells was analyzed by Western blotting (a, n = 5; b and c, n = 3). BC - bead control. [file 12943_2023_1792_MOESM3_ESM.tif]

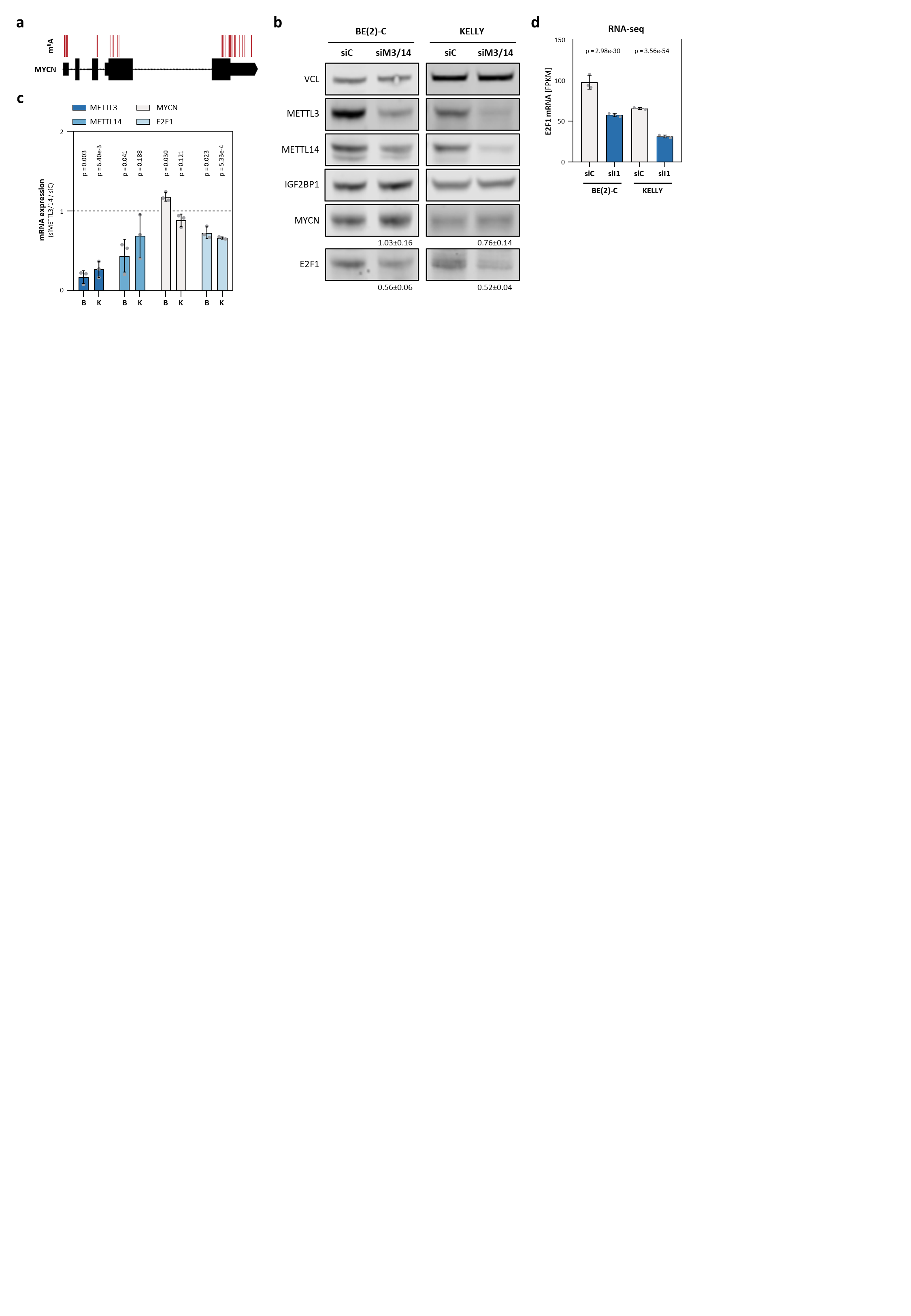

Supplement: Supplementary file 4 — Additional file 4: Supplementary Fig. 4. Regulation of MYCN by IGF2BP1 is largely m6A-independent. (a) N6-Methyladenosine (m6A) modification profile of MYCN mRNA. (b, c) Western blot (b; BE(2)-C, n = 3; KELLY, n = 4) and RT-qPCR (c; n = 3) analyses of MYCN expression upon co-depletion of the key mRNA m6A-methyltransferase complex METTL3/14 (siM3/14) compared to control knockdown (siC). (d) RNA-seq data of E2F1 expression in BE(2)-C and KELLY upon transient control (siC) and IGF2BP1 (siI1) knockdown (n = 3). B - BE(2)-C, K – KELLY. [file 12943_2023_1792_MOESM4_ESM.tif]

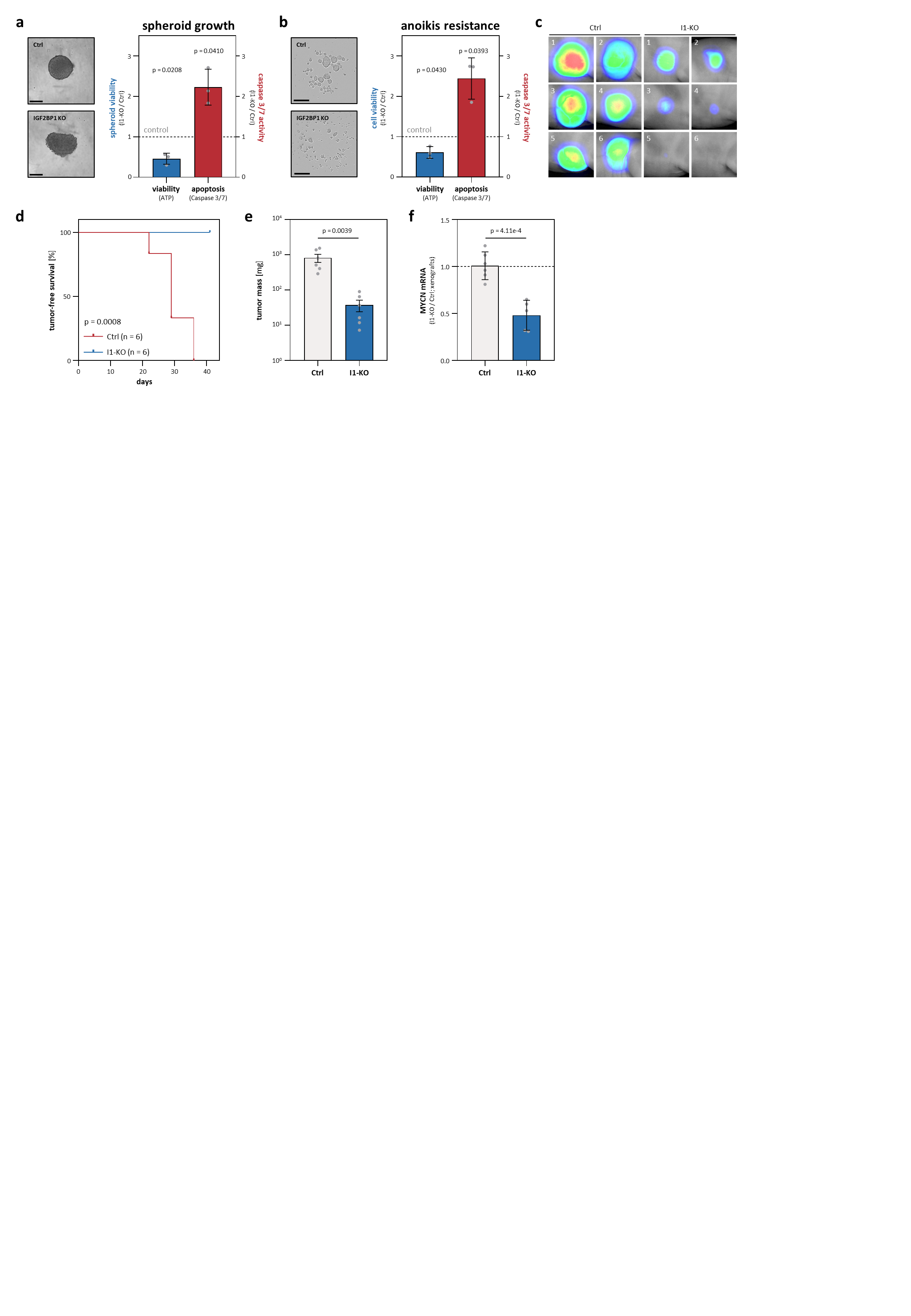

Supplement: Supplementary file 5 — Additional file 5: Supplementary Fig. 5. Oncogenic potential of IGF2BP1 is conserved in nMNA neuroblastoma cell and xenograft model. (a, b) The viability and caspase3/7 activity of parental (Ctrl) and I1-KO NBL-S was analyzed in spheroid growth (a; n = 3) and anoikis resistance studies (b; n = 3; bars a, 200 µm; b, 400 µm). (c-e) Tumor growth (n = 6) of Ctrl and I1-KO NBL-S s.c. xenografts was monitored by non-invasive infrared imaging (c), tumor-free survival (d), and final tumor mass (e). (f) RT-qPCR analysis of MYCN mRNA levels in excised parental xenograft tumors (n = 6) and residual non-palpable I1-KO tumors (n = 5). [file 12943_2023_1792_MOESM5_ESM.tif]

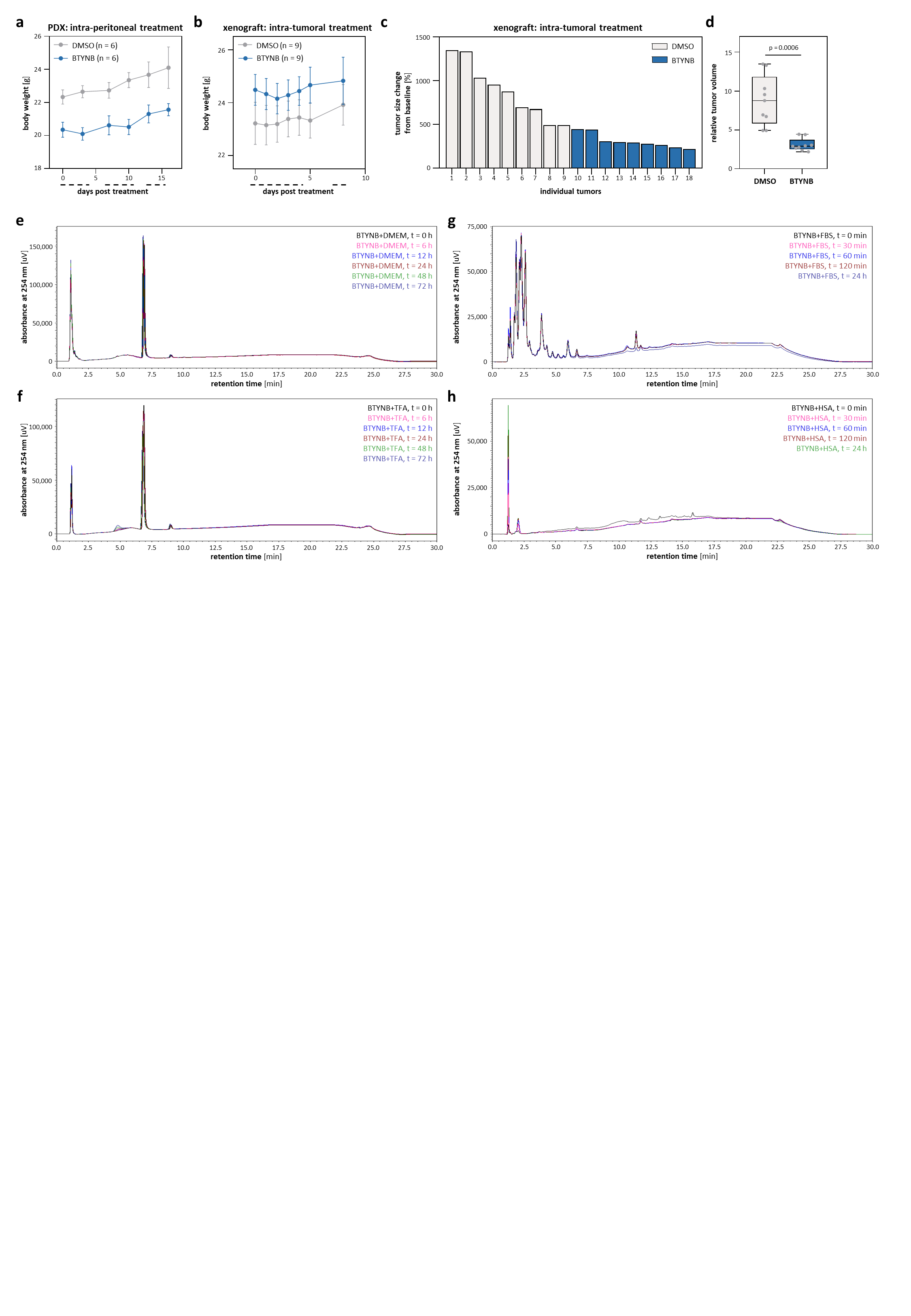

Supplement: Supplementary file 6 — Additional file 6: Supplementary Fig. 6. BTYNB is stable, has minimal effect on mouse body weight, but high serum protein binding capacity. (a, b) Body weight analysis of (a, n = 6; b, n = 9) mice with s.c. PDX (a) or BE(2)-C xenograft tumors (b) treated i.p. (a) or i.t. (b) with DMSO (grey) or BTYNB (blue). Treatment cycles are indicated by dashed lines below the x-axis. (c) Waterfall plot showing the percentage difference in tumor size from treatment day 0 to day 8 in BE(2)-C xenograft mice treated i.t. with DMSO (beige) or BTYNB (blue; n = 9). (d) Final relative tumor volume of s.c. BE(2)-C xenografts tumors treated with DMSO or BTYNB (n = 9). (e-h) Superimposed HPLC chromatograms for BTYNB stability in DMEM (e), under acidic conditions (f) and after incubation with FBS (g) or HSA (h) at different time points. [file 12943_2023_1792_MOESM6_ESM.tif]

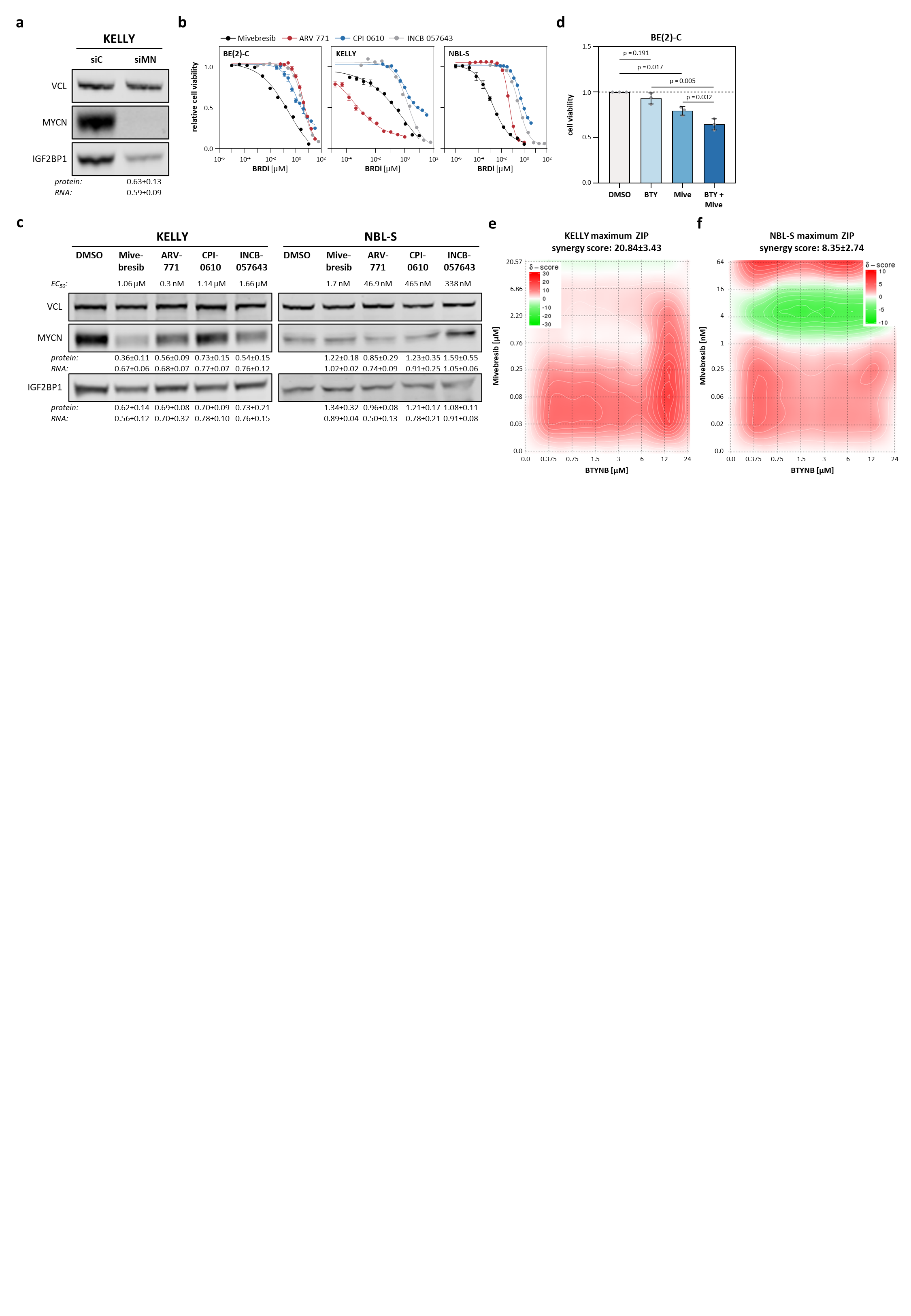

Supplement: Supplementary file 7 — Additional file 7: Supplementary Fig. 7. MYCN-driven and BRDi-directed inhibition of IGF2BP1 synthesis is conserved in neuroblastoma cell models. (a) Western blot (n = 3) and RT-qPCR (n = 6) analysis of IGF2BP1 expression upon MYCN (siMN) compared to control knockdown (siC) in KELLY. (b) BRD inhibitor response curve in BE(2)-C, KELLY and NBL-S (n = 4). (c) Western blot (KELLY, n = 3; NBL-S, n = 4) and RT-qPCR (n = 3) analysis of MYCN and IGF2BP1 expression after treatment of KELLY or NBL-S with indicated BRD inhibitors. (d) BE(2)-C cell viability upon treatment with optimal synergy concentrations (750 nM BTYNB, 10 nM Mivebresib) alone or in combination. BTY - BTYNB, Mive - Mivebresib. (e, f) Relief plot showing the ZIP synergy for combined treatment of BTYNB and Mivebresib in KELLY (e) and NBL-S (f; n = 3). [file 12943_2023_1792_MOESM7_ESM.tif]

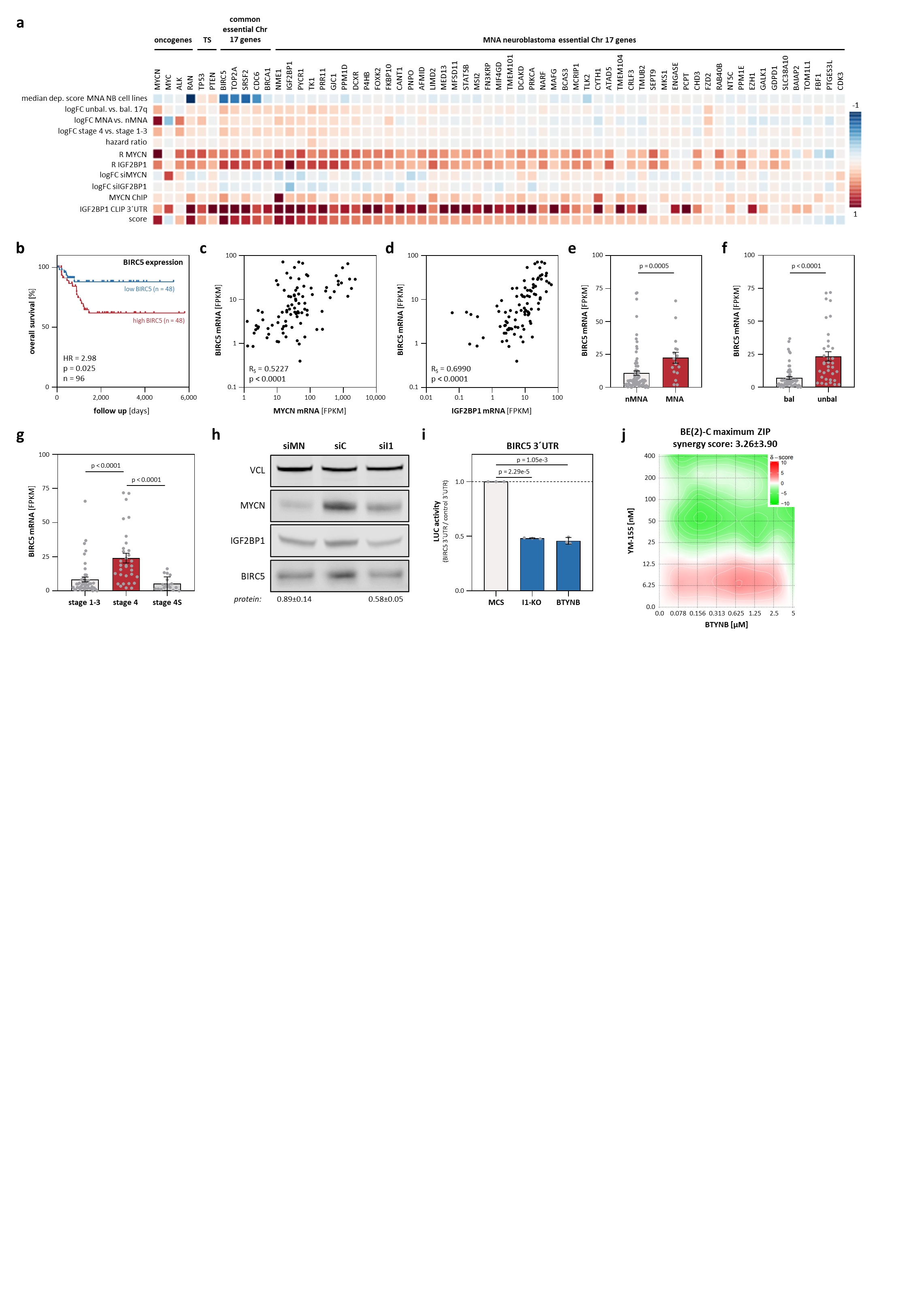

Supplement: Supplementary file 8 — Additional file 8: Supplementary Fig. 8. MYCN/IGF2BP1-driven upregulation of BIRC5 is conserved in neuroblastoma models and primary human disease. (a) Heatmap representing the identification of potential IGF2BP1 and MYCN downstream targets. Neuroblastoma and common essential genes located on Chr 17q were analyzed as described (for details refer to the online method section). Data of each row were scaled to range 0 and 1 (ChIP and CLIP) or -1 and 1 (all other). (b) Kaplan-Meier survival analysis of human neuroblastoma by BIRC5 expression (median cut-off). (c, d) Spearman correlation analyses of BIRC5 with MYCN (c) or IGF2BP1 (d) mRNA expression in human neuroblastoma tumor samples. (e-g) Expression of BIRC5 in neuroblastoma separated by MYCN status (e), Chr 17 balance status (f) and INSS tumor stage (g). (h) Western blot analysis of BIRC5 expression upon transient MYCN (siMN) or IGF2BP1 (siI1) compared to control (siC) knockdown in KELLY (n = 5). (i) BIRC5 3’UTR reporter activity in I1-KO versus Ctrl and BTYNB- versus DMSO-treated KELLY (n = 3). (j) Relief plot showing the ZIP synergy for combined treatment of BTYNB and YM-155 in BE(2)-C (n = 3). nMNA - MYCN non-amplified, MNA - MYCN-amplified, bal - balanced Chr 17, unbal - unbalanced Chr 17, TS - tumor suppressor. [file 12943_2023_1792_MOESM8_ESM.tif]

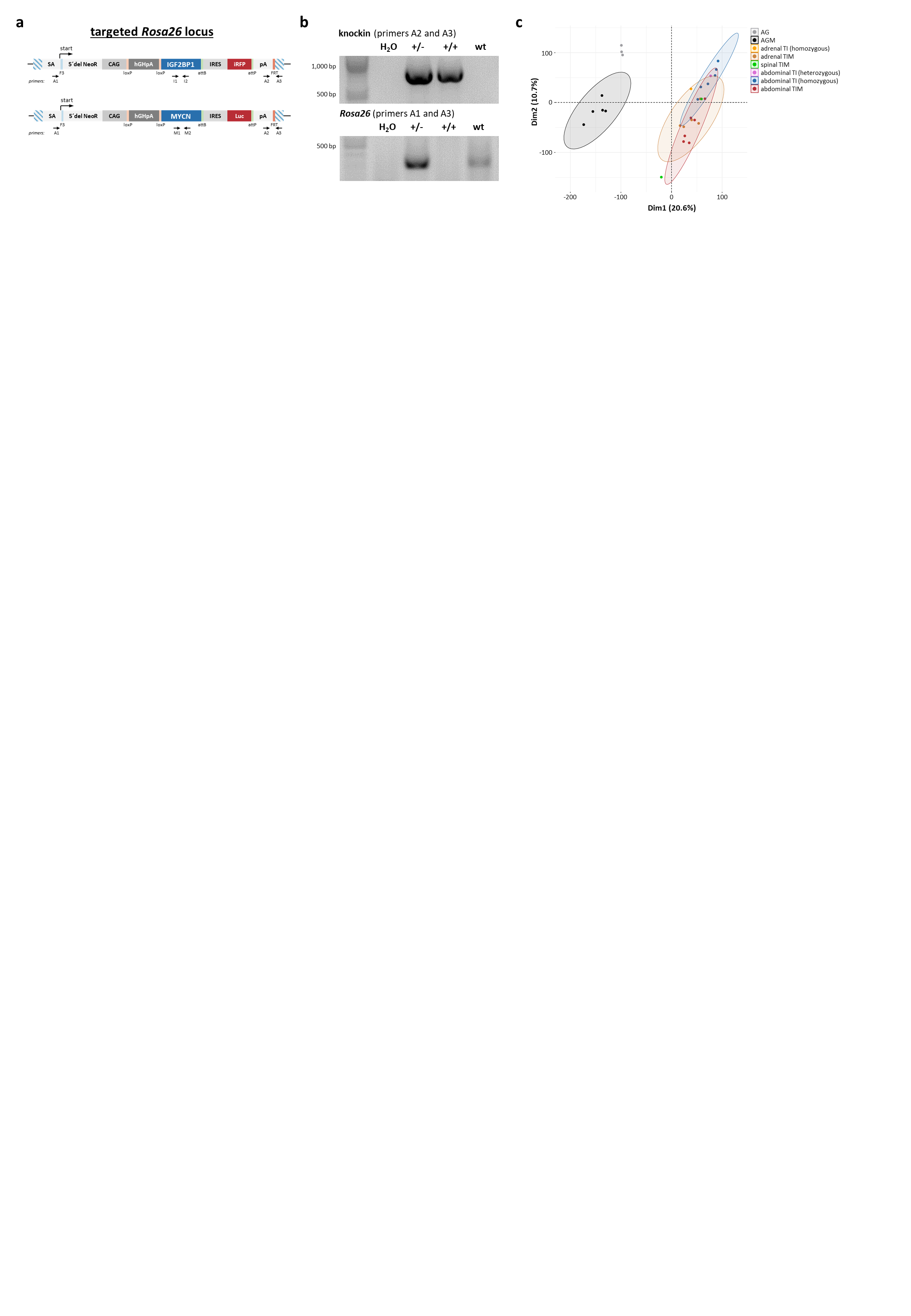

Supplement: Supplementary file 9 — Additional file 9: Supplementary Fig. 9. Expression of transgenic tumors is largely location-independent. (a) Schematic overview of the LSL-transgene expression of IGF2BP1 and iRFP or MYCN and luciferase respectively from the Rosa26 locus. Location of primers used for genotyping (A1, A2, A3, I1, I2, M1, M2) are indicated. Splice acceptor site (SA), neomycin resistance (5´del NeoR), the synthetic promoter (CAG), transcriptional stop cassette made of the human growth hormone polyadenylation signal (hGHpA), transgene open reading frame (blue), internal ribosomal entry site (IRES), iRFP or luciferase open reading frame (red), polyadenylation signal (pA). (b) Representative genotyping PCR validating the transgene knockin (top, primers A2 and A3) and the wildtype Rosa26 locus (bottom, primers A1 and A3) in heterozygous (+/-), homozygous (+/+) and wildtype (wt) mice. (c) PCA of all sequenced adrenal glands and tumors split by their location. AG - wildtype adrenal gland, AGM - adrenal gland from R26MYCN, TI - tumor from R26IGF2BP1, TIM - tumor from R26IGF2BP1/MYCN. [file 12943_2023_1792_MOESM9_ESM.tif]

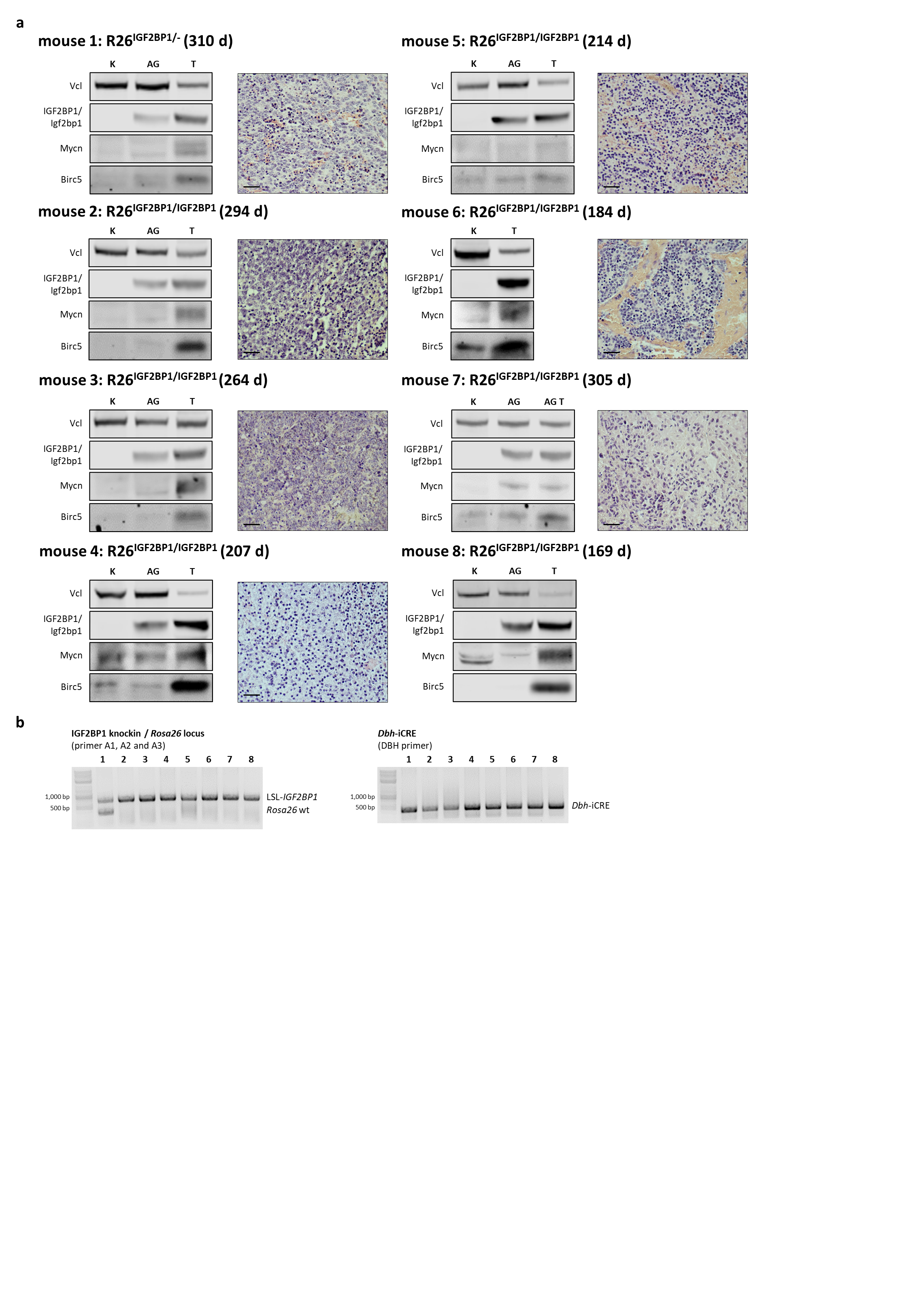

Supplement: Supplementary file 10 — Additional file 10: Supplementary Fig. 10. Summary and validation of analyzed R26IGF2BP1 tumors. (a) For each analyzed LSL-IGF2BP1 mouse with obvious signs of tumor burden, expression of IGF2BP1/Igf2bp1, Mycn and Birc5 was validated by Western blotting (left panels, n = 1). Time until termination criteria is reached are indicated in brackets. Mouse 1-6 and 8 had abdominal tumors, whereas mouse 7 had an adrenal gland tumor. HE staining was performed on excised tumors and representative images are shown in right panels (bars 200 µm). (b) Genotyping PCR performed as in Supplementary Figure 10 validated IGF2BP1 transgene (LSL-IGF2BP1), Rosa26 wildtype locus (Rosa26 wt) and Dbh-iCRE. K - kidney, AG - adrenal gland, T - tumor, AG T - adrenal gland tumor. [file 12943_2023_1792_MOESM10_ESM.tif]

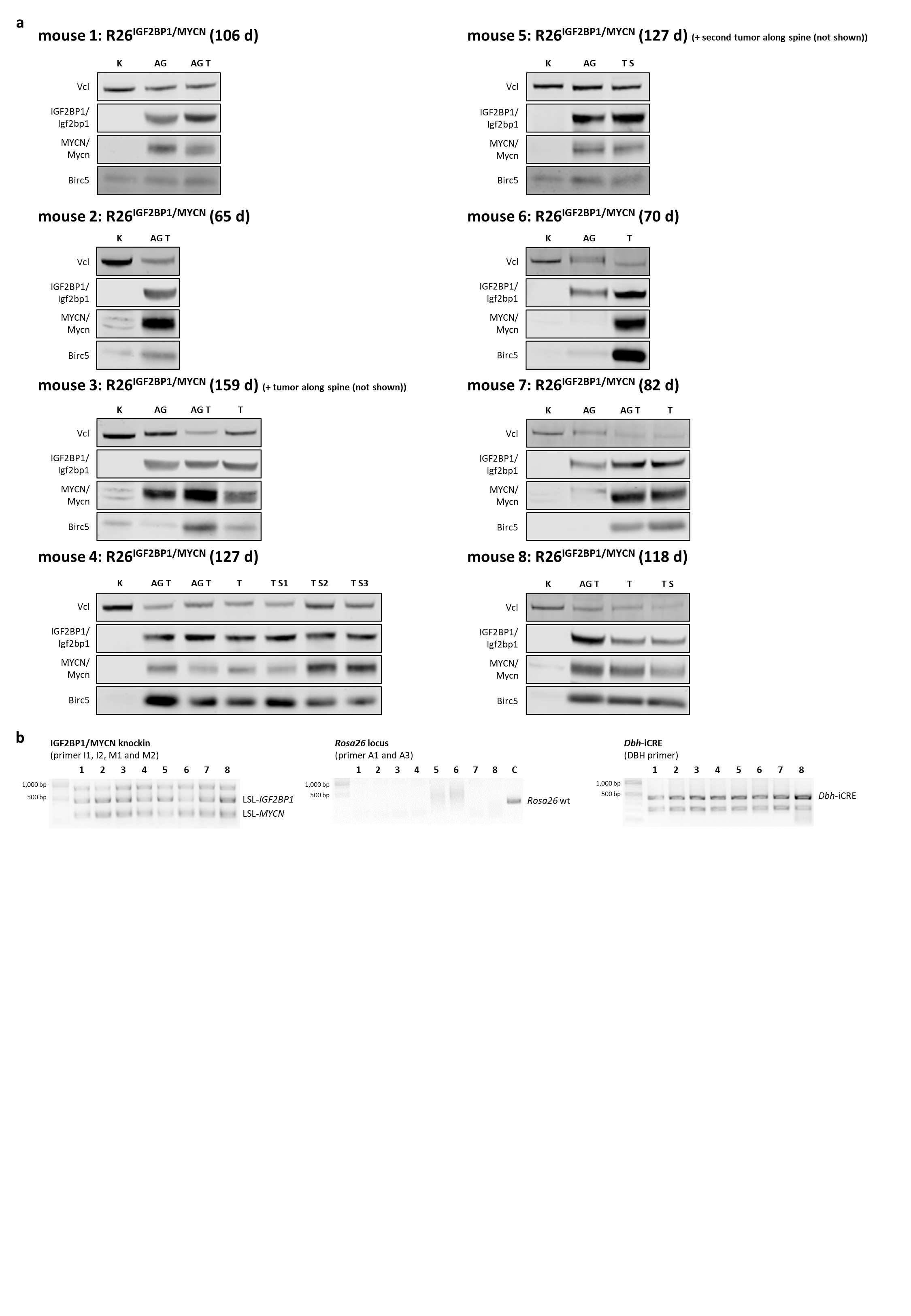

Supplement: Supplementary file 11 — Additional file 11: Supplementary Fig. 11. Summary and validation of analyzed R26IGF2BP1/MYCN tumors. (a) For each analyzed LSL-IGF2BP1/MYCN mouse with obvious signs of tumor burden, expression of IGF2BP1/Igf2bp1, MYCN/Mycn and Birc5 was validated by Western blotting (n = 1). Time until termination criteria is reached are indicated in brackets. (b) Genotyping PCR performed as in Supplementary Figure 10 validated IGF2BP1 and MYCN transgene (LSL-IGF2BP1, LSL-MYCN), absence of Rosa26 wildtype locus (Rosa26 wt) and Dbh-iCRE. K - kidney, AG - adrenal gland, T - tumor, AG T - adrenal gland tumor, T S - tumor along spine, C - control wildtype mouse. [file 12943_2023_1792_MOESM11_ESM.tif]

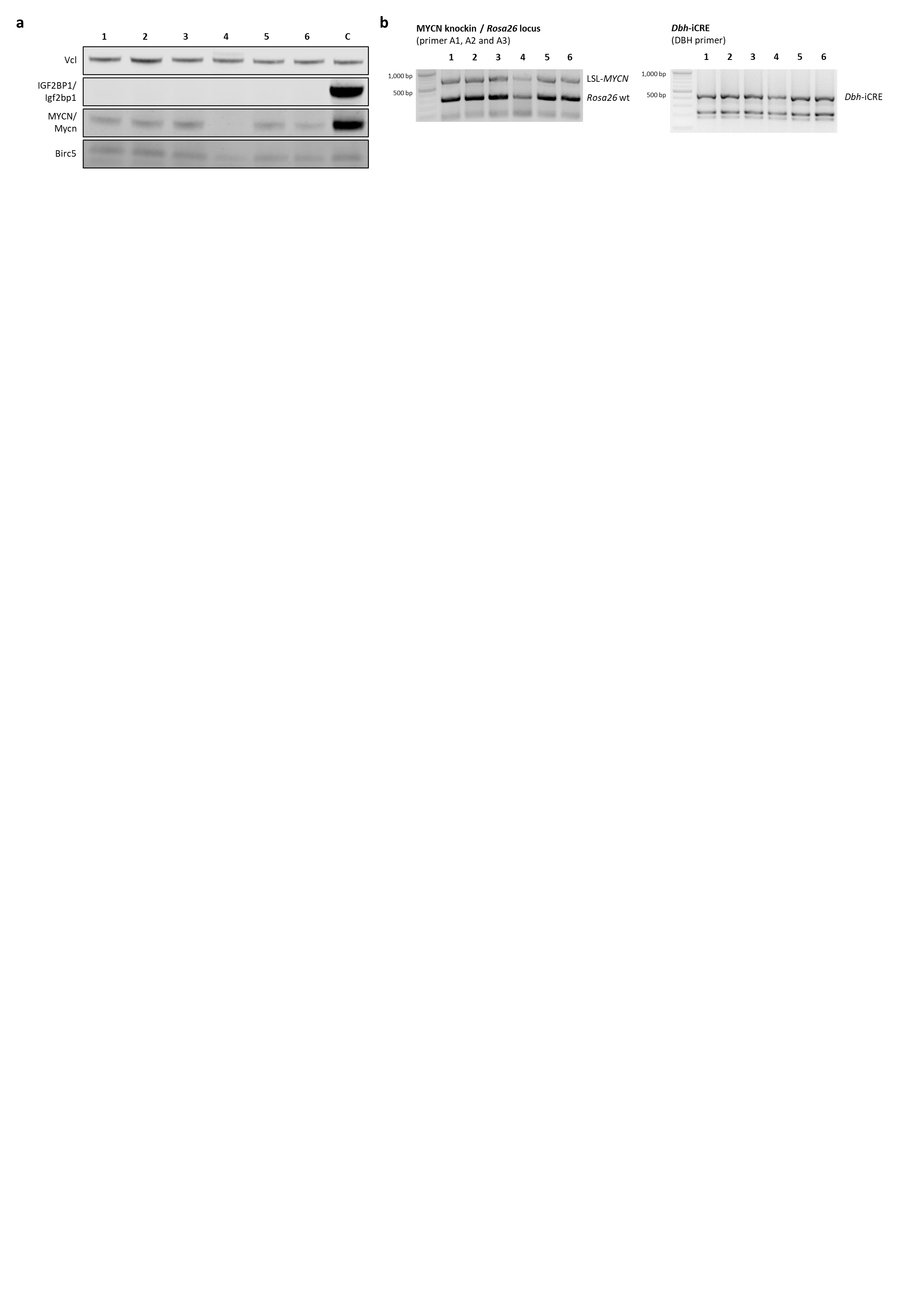

Supplement: Supplementary file 12 — Additional file 12: Supplementary Fig. 12. Summary and validation of analyzed R26MYCN adrenal glands. (a) For each analyzed LSL-MYCN mouse expression of Igf2bp1, MYCN/Mycn and Birc5 was validated by Western blotting (n = 1). Each mice were culled after 365 days. As control (C) served an adrenal gland from a R26IGF2BP1/MYCN mouse. (b) Genotyping PCR validated MYCN transgene (LSL-MYCN), Rosa26 wildtype locus (Rosa26 wt) and Dbh-iCRE. [file 12943_2023_1792_MOESM12_ESM.tif]

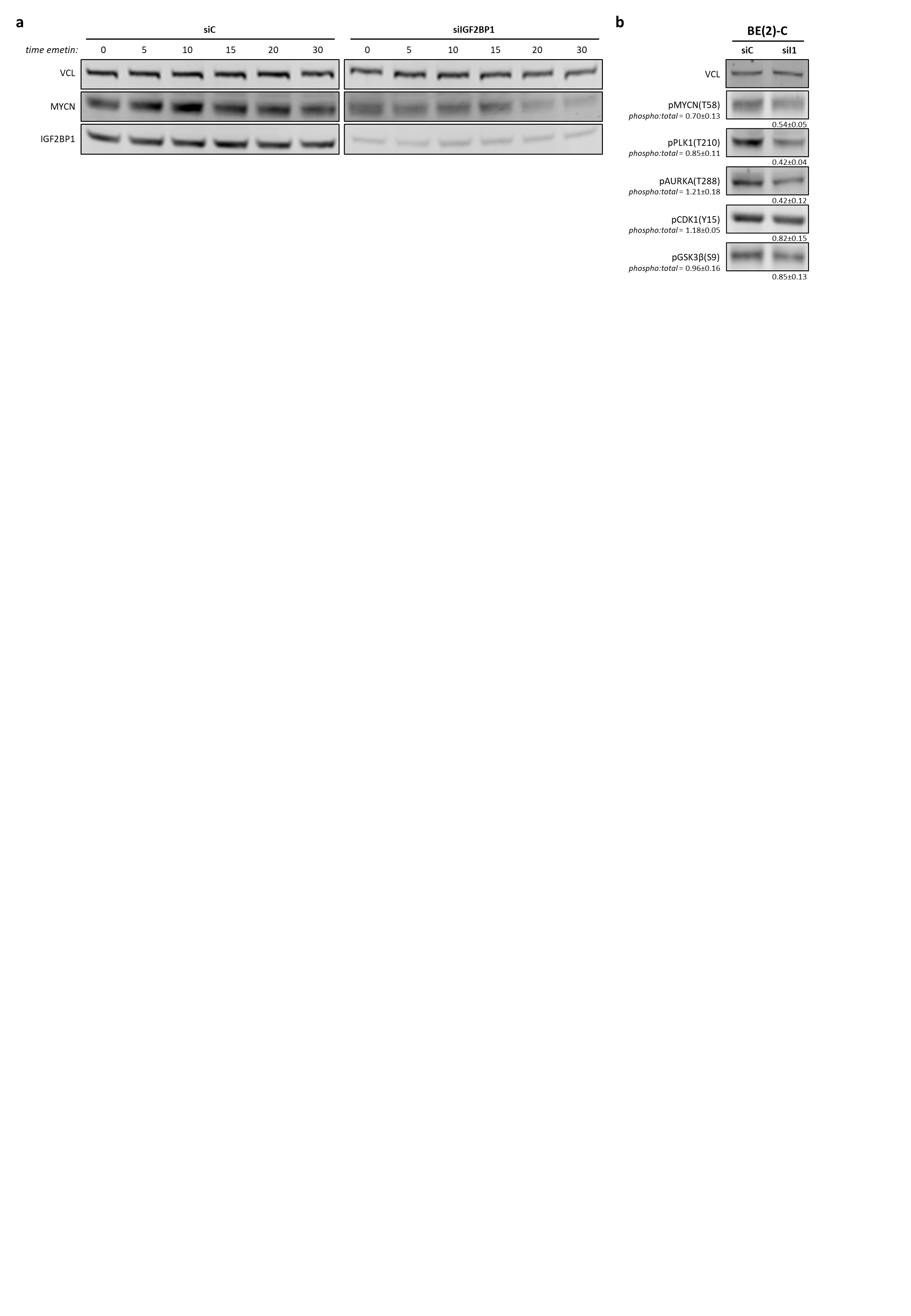

Supplement: Supplementary file 13 — Additional file 13: Supplementary Fig. 13. IGF2BP1 influences MYCN protein turnover. (a) Western blot (n = 3) analysis of MYCN protein decay upon transient IGF2BP1 (siIGF2BP1) or control (siC) knockdown in TET21N. Treatment time with emetin in minutes are indicated. (b) Western blot (n = 3) analysis of indicated phosphoproteins upon IGF2BP1 (siI1) compared to control (siC) knockdown in BE(2)-C. Relative changes of phosphorylation signals to total protein level are indicated. [file 12943_2023_1792_MOESM13_ESM.tif]

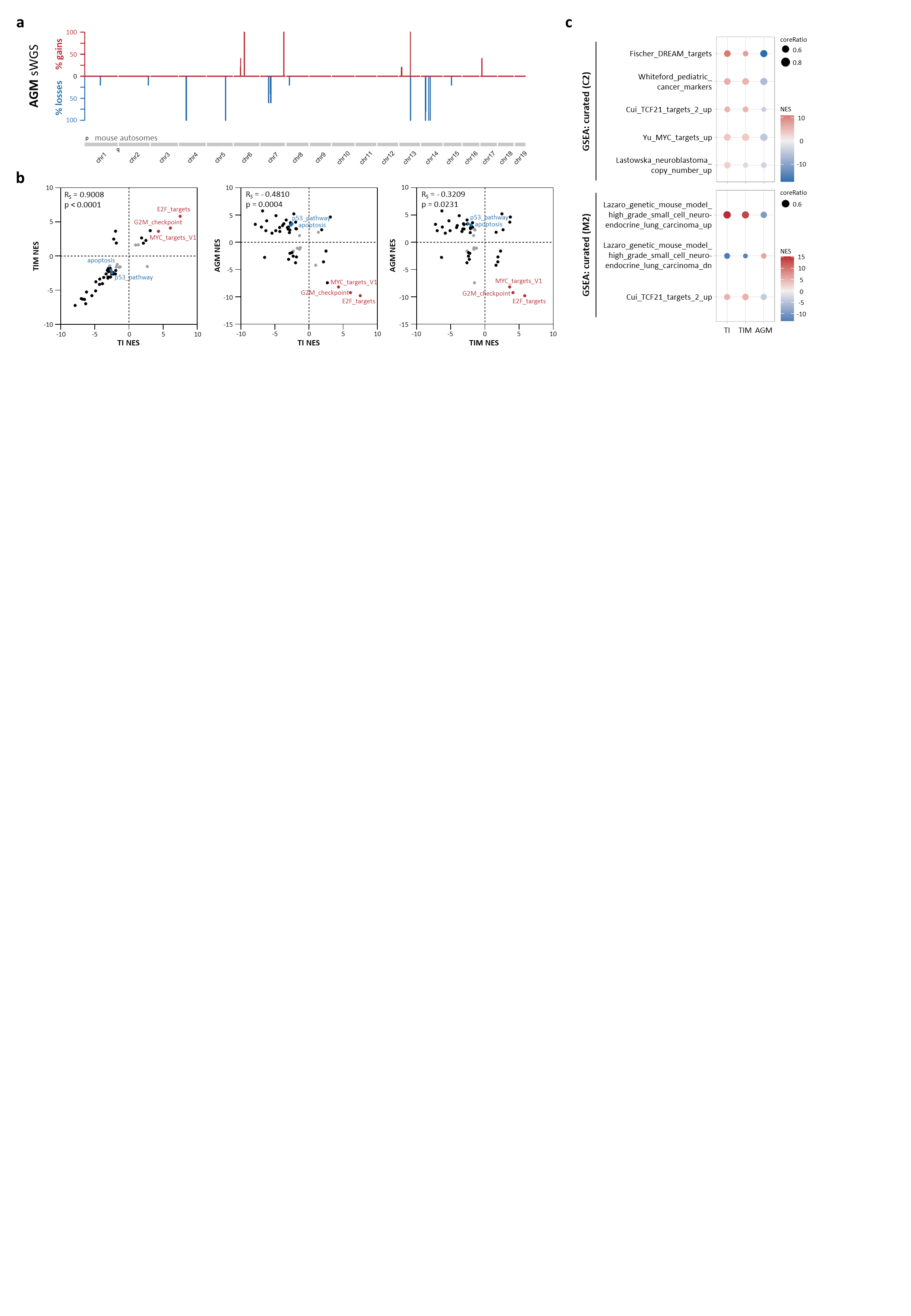

Supplement: Supplementary file 14 — Additional file 14: Supplementary Fig. 14. Genomic aberration are nearly absent in R26MYCN adrenal glands. (a) Frequency (%) of DNA copy number gains (red) and losses (blue) for murine chromosome 1 to 19 in R26MYCN compared to wildtype adrenal glands. (b) Correlation of NES values of hallmark gene sets between TI and TIM (left) as well as tumors and AGM (middle, right). Non-significant gene sets are depicted in grey. (c) Selected gene sets from curated human (C2) and mouse (M2) collection in R26IGF2BP1 (TI), R26IGF2BP1/MYCN (TIM) or R26MYCN (AGM) mice based on GSEA. [file 12943_2023_1792_MOESM14_ESM.tif]
